# Heterologous expression in *Tritrichomonas foetus *of functional *Trichomonas vaginalis *AP65 adhesin

**DOI:** 10.1186/1471-2199-6-5

**Published:** 2005-03-04

**Authors:** Ashwini S Kucknoor, Vasanthakrishna Mundodi, JF Alderete

**Affiliations:** 1 Department of Microbiology, University of Texas Health Science Center at San Antonio, 7703, Floyd Curl Dr. San Antonio, TX, 78229-3900 USA

## Abstract

**Background:**

Trichomonosis, caused by *Trichomonas vaginalis*, is the number one, nonviral sexually transmitted infection that has adverse consequences for the health of women and children. The interaction of *T. vaginalis *with vaginal epithelial cells (VECs), a step preparatory to infection, is mediated in part by the prominent surface protein AP65. The bovine trichomonad, *Tritrichomonas foetus*, adheres poorly to human VECs. Thus, we established a transfection system for heterologous expression of the *T. vaginalis *AP65 in *T. foetus*, as an alternative approach to confirm adhesin function for this virulence factor.

**Results:**

In this study, we show stable transfection and expression of the *T. vaginalis ap65* gene in *T. foetus *from an episomal pBS-*ap65-neo* plasmid. Expression of the gene and protein was confirmed by RT-PCR and immunoblots, respectively. AP65 in transformed *T. foetus *bound to host cells. Specific mAbs revealed episomally-expressed AP65 targeted to the parasite surface and hydrogenosome organelles. Importantly, surface-expression of AP65 in *T. foetus *paralleled increased levels of adherence of transfected bovine trichomonads to human VECs.

**Conclusion:**

The *T. vaginalis *AP65 adhesin was stably expressed in *T. foetus*, and the data obtained using this heterologous system strongly supports the role of AP65 as a prominent adhesin for *T. vaginalis*. In addition, the heterologous expression in *T. foetus *of a *T. vaginalis *gene offers an important, new approach for confirming and characterizing virulence factors.

## Background

The colonization of the urogenital tract of humans by the protozoan parasite *Trichomonas vaginalis *is responsible for trichomonosis [1], the most prevalent, non-viral sexually transmitted infection worldwide. Despite an estimated 8 million new cases per year in the United States alone [2], this health disparities disease [3] remains poorly studied. *T. vaginalis *infection is associated with adverse health consequences to both men and women, including infertility [4,5], atypical pelvic inflammatory disease [6], and increased HIV transmission [7,8]. Trichomonosis is also associated with preterm birth, low birth weight infants [9], predisposition to development of cervical neoplasia [10] in women and non-gonococcal urethritis [11] and chronic prostatitis [12] in men. *T. vaginalis *adherence to host VECs, a step preparatory to infection [13], is complex and involves four surface protein adhesins. Three adhesins studied to date at the molecular level share identity with metabolic enzymes of the hydrogenosome organelle [14,15]. Thus, the proteins exhibit functional diversity based on cellular location. AP65 is a prominent *T. vaginalis *adhesin that is targeted both to the surface and hydrogenosomes, and this compartmentalization is in part modulated by iron [15]. Further, the surface placement of AP65 is increased upon contact with host cells [15]. Recently, silencing of *ap65 *gene expression in *T. vaginalis *with antisense reinforced the role of AP65 as a major adhesion [16].

The related *Tritrichomonas foetus *is a causal agent of bovine fetal wastage [17,18]. Adherence by *T. foetus *to bovine VECs appears equally complex as that seen for *T. vaginalis *except that in this case, adherence involves glycoconjugates [19,20]. Interestingly, the interaction between *T. foetus *organisms and VECs is both cell- and species-specific [21,22]. For example, *T. foetus *attaches to bovine VECs, MDCK and HeLa epithelial cells, which have been used previously for studies on the host-parasite interaction. However, the bovine trichomonads have low background levels of adherence to human VECs. This may suggest that the phylogenetically-related *T. vaginalis *and *T. foetus *trichomonads differ in mechanisms of host parasitism and cytopathogenicity. We hypothesized that, given the different mechanisms of host VEC adherence by *T. foetus *and *T. vaginalis*, the heterologous expression in *T. foetus *of the *T. vaginalis *AP65 could be an alternative approach to confirm the function of AP65 as an adhesin.

Importantly, no cross-hybridization and immuno-crossreactivity with *ap65 *and mAbs to AP65, respectively, were evident in *T. foetus*. We now show the stable expression in *T. foetus *of a single copy of the *T. vaginalis ap65 *gene encoding an ~65-kDa protein [23]. The stable transfectants expressed functional AP65 on the *T. foetus *surface. These findings importantly illustrate the utility of a stable transfection system for heterologus expression in *T. foetus *of *T. vaginalis *virulence genes.

## Results

### Low levels of adherence to immortalized human MS-74 VECs and lack of ap65 by *T. foetus*

**Figure 1:**
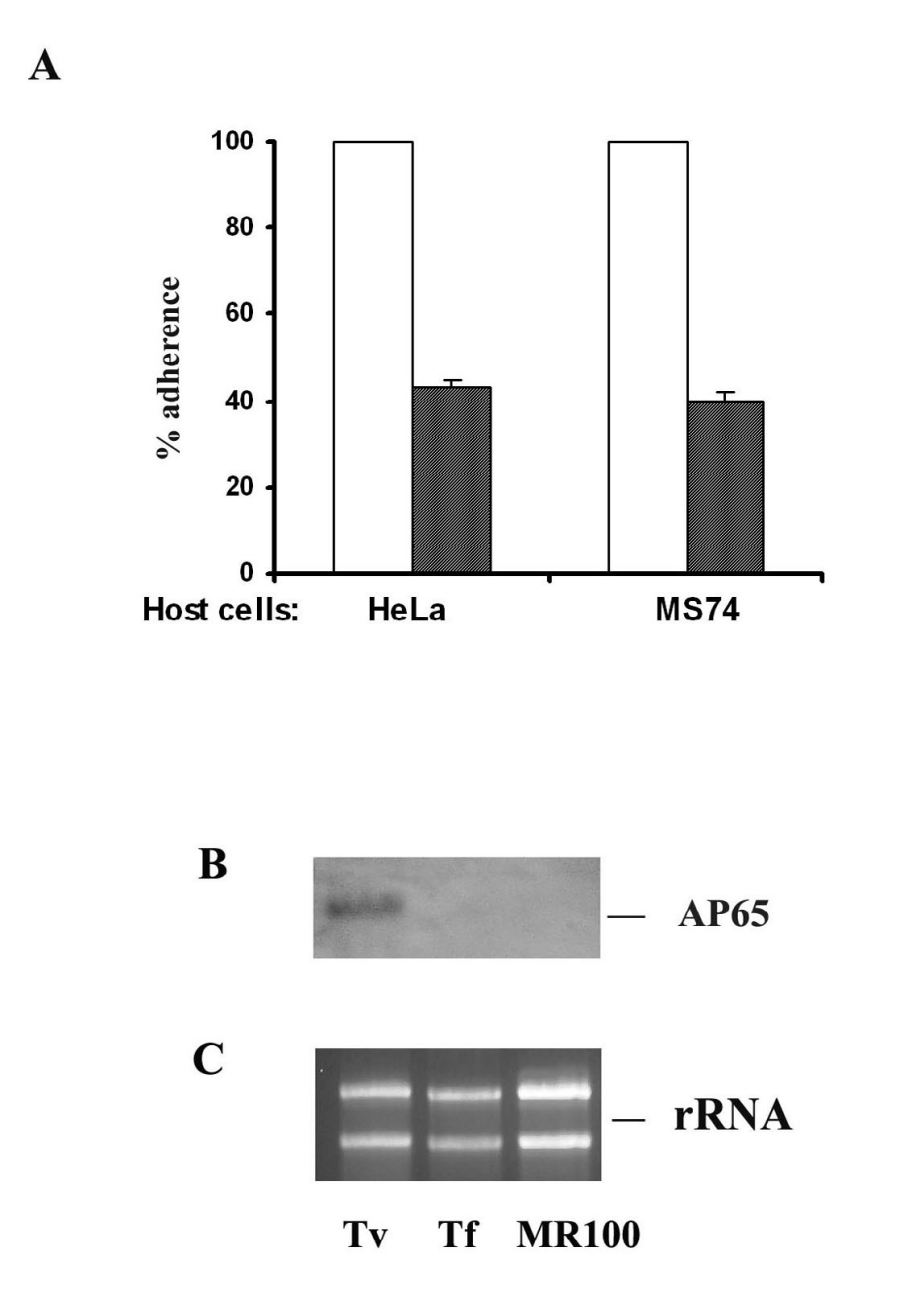
***Tritrichomonas foetus *has lower levels of adherence to HeLa epithelial cells and immortalized human MS-74 VECs and has no detectable *ap65 *adhesin gene**. (A) Bar graph showing the mean level of adherence by *T. foetus *(shaded bars) derived from four independent adherence experiments, each of which was performed with quadruplicate samples. Adherence levels of *T. vaginalis *(open bars) were normalized to 100% for comparative purposes. (B) Northern analysis to detect *ap65 *mRNA amounts was performed using 10 μg of total RNA per lane separated on 1.2% agarose-formaldehyde gels and transferred to Hybond-P membranes. The blot was probed with DIG-labelled *ap65*, which hybridized to an ~2-kb *ap65 *transcript in *T. vaginalis*, as before [16]. No similar signal was detected in *T. foetus *and the drug resistant MR100 *T. vaginalis *used as a negative control [15]. (C) Ethidium bromide (EtBr)-stained RNA gel showing the rRNA bands. Equal loading of RNA in all the lanes is evident by similar amounts of the rRNA bands, which served as internal controls for the Northern blot in part B.

We wanted to perform comparative binding experiments between *T. vaginalis *and *T. foetus *to epithelial cells used in adherence assays [15]. Given the fact that *T. foetus *exhibits host tissue- and cell-specificity [24], it was not surprising to find the lower levels of adherence by *T. foetus *to HeLa epithelial cells and immortalized human MS-74 VECs. As shown in representative experiment in Figure 1, at best only 40% levels of adherence were seen for *T. foetus *compared to *T. vaginalis*.

We next wanted to know if *T. foetus *had mRNA that hybridized to *ap65 *used as a probe. As shown in Figure 1B, no RNA band was detected by Northern analysis for *T. foetus *(lane labeled Tf). In contrast, a band of ~2-kb was readily hybridized by total RNA of *T. vaginalis*. MR100, the drug-resistant *T. vaginalis *that is unable to adhere to host cells as shown recently also had no detectable *ap65 *transcript, as a control [15]. The equivalent amounts of rRNA bands seen in Figure 1C illustrate that similar amounts of RNA were added to the lanes used in the Northern blots (Figure 1B).

### Episomal expression in *T. foetus* of *ap65*

**Figure 2:**
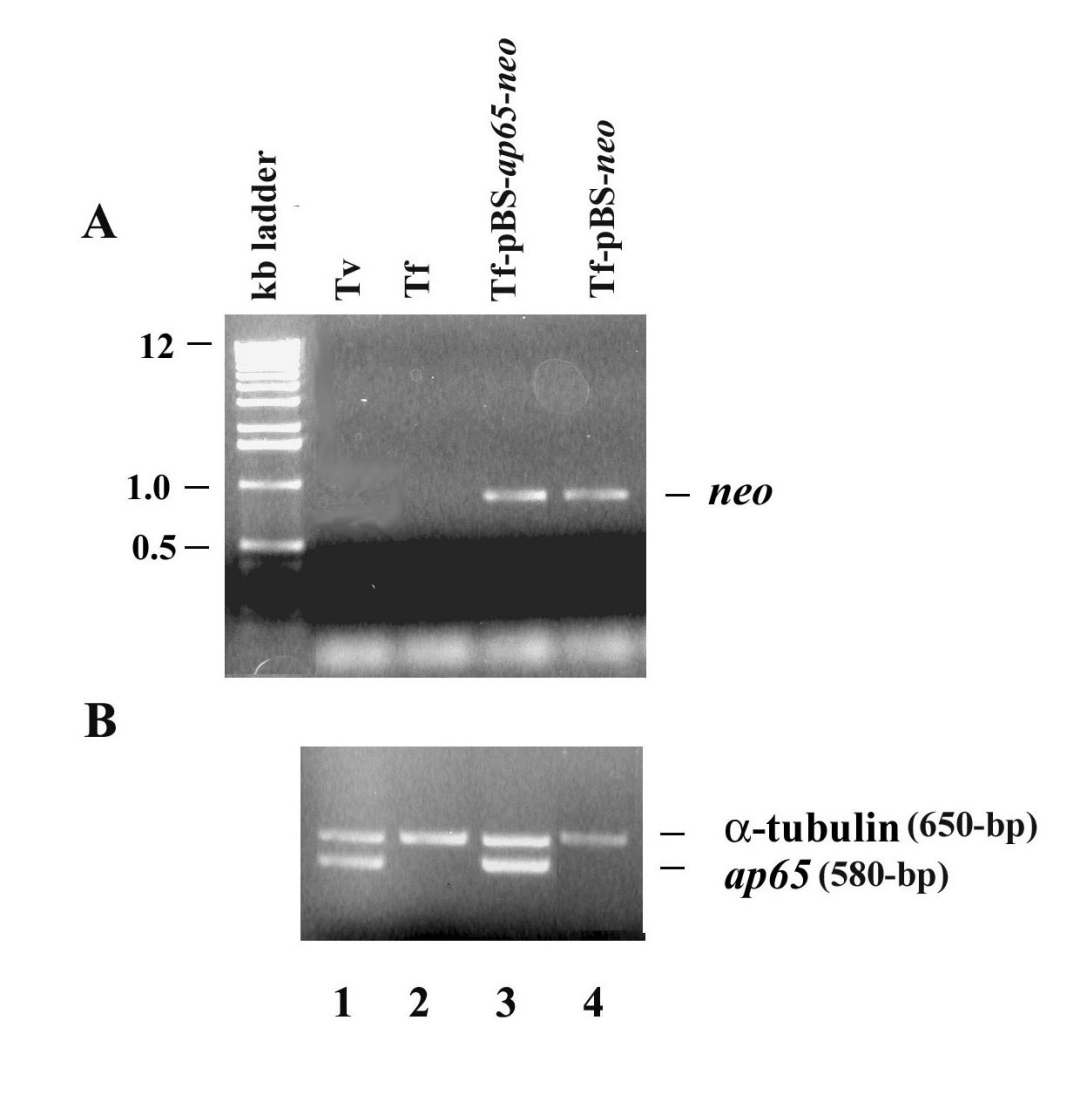
**Transfection and episomal expression of *ap65 *in *T. foetus***. (A) Agarose gel stained with EtBr shows results of a PCR reaction to amplify the 795-bp *neo *gene from transfected *T. foetus *total genomic DNA. The *neo *gene PCR product is detected only in *T. foetus *transfected with pBS-*ap65-neo *(lane 3) and pBS-*neo *(lane 4), but not the wild type *T. foetus *(Tf; lane 2) and control *T. vaginalis *(Tv; lane 1). (B) Agarose gel stained with EtBr shows RT-PCR products for the *ap65 *transcript in wt *T. foetus *(lane 1), *T. vaginalis *(lane 2), *T. foetus *transfected with pBS-*ap65-neo *(lane 3), and *T. foetus *transfected with the pBS-*neo *plasmid as a negative control. RT-PCR was performed using primers to amplify a 580-bp region of the *ap65 *gene and a 650-bp region of the a-tubulin gene used as an internal control.

The expression plasmid pBS-*ap65-neo *contains the neomycin (*neo*) gene, and as done recently, the transfectants were selected for Geneticin resistance at 100μg/ml [16]. As shown in Figure 2A, the presence of plasmid in transfectants was verified by PCR amplification of the *neo *coding region. A band of 750-bp was detected in the transfectants corresponding to pBS-*ap65-neo *(lane3) and pBS-*neo *(lane 4). Not unexpectedly, no PCR products were obtained using *T. vaginalis *(lane 1) and *T. foetus *(lane 2).

We then checked for the episomal gene expression by means of RT-PCR. The PCR products following reverse transcription were separated on 1% agarose gels. Figure 2B confirms the presence of an *ap65 *band corresponding to 580-bp amplified from the transfectants with pBS-*ap65-neo *(lane 3), confirming the expression of *ap65 *from the plasmid. The same band is also seen in *T. vaginalis *(lane 1). As an internal control in these experiments, the common band of 650-bp seen in all lanes corresponds to the *a-tubulin *gene. This control was important to show the similar amounts of RNA used for RT-PCR in all samples. These results demonstrate that the episomal plasmid with the *T. vaginalis ap65 *gene is successfully expressed in transfected *T. foetus*.

### *T. vaginalis* AP65 and AP65-HA are expressed in *T. foetus*, and episomal AP65 has function

**Figure 3:**
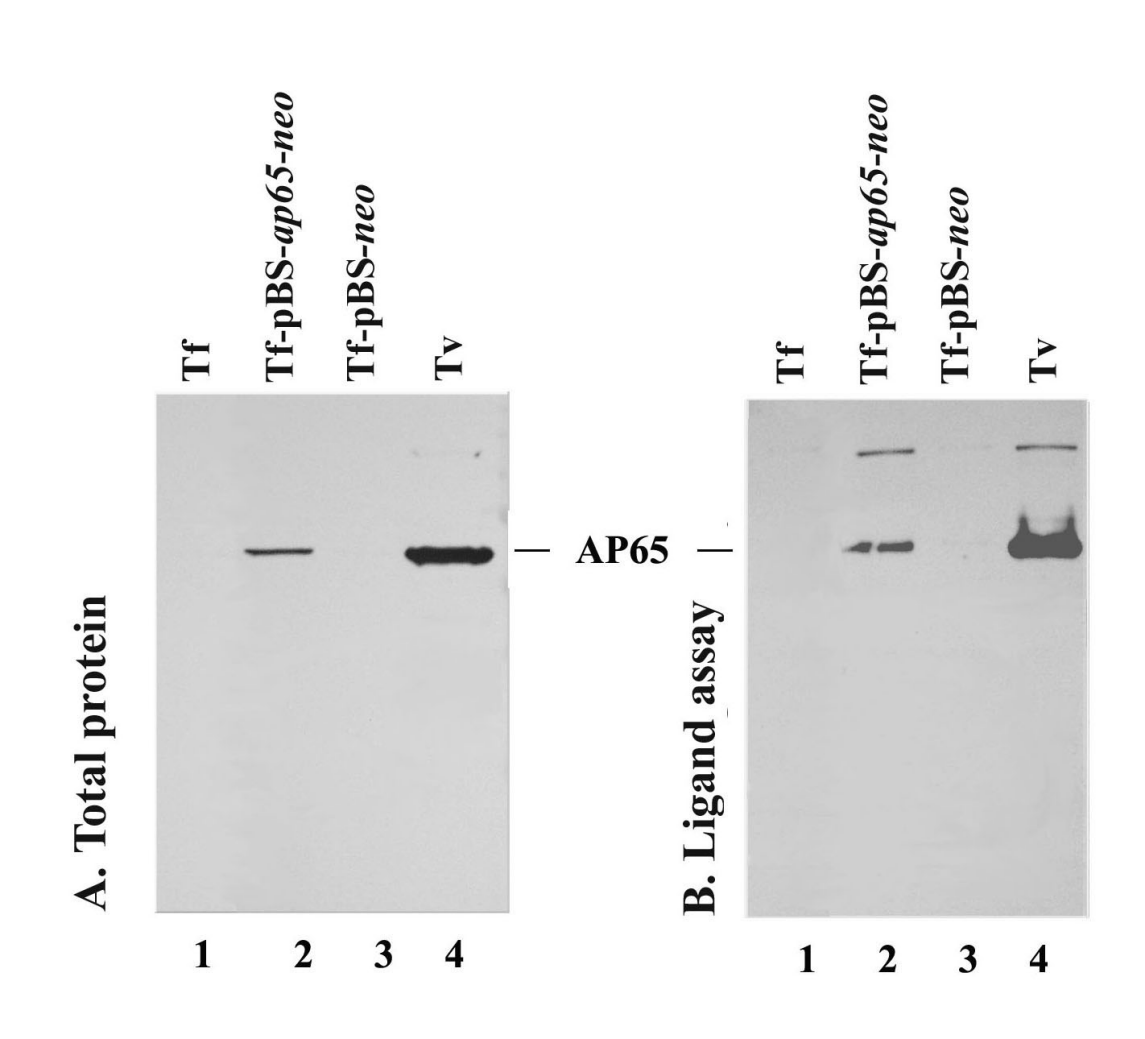
**Monoclonal antibody (mAb) detects AP65 expressed in pBS-*ap65-neo *(Parts A and B) and pBS-*ap65-HA-neo *(Part C) transfected *T. foetus *parasites**. (A) Total proteins from 10^7 ^parasites were separated on 10% SDS-PAGE and blotted onto Hybond-P membranes for probing with mAb DM116 specific for AP65, as before (Garcia *et al*., 2003). The AP65 protein was readily detected in total protein blots of transfected *T. foetus *(lane 2) and *T. vaginalis *(lane 4), but not in wt *T. foetus *(lane 1) and in *T. foetus *transfected with the control pBS-*neo *plasmid (lane 3). The migration of detected AP65 was ~65-kDa as expected based on molecular weight standards. (B) A ligand assay showing AP65 binds to immortalized human MS-74 VECs, as before (Garcia *et al*., 2003). Parasites bound to VECs were solubilized and electrophoresed as above for blotting onto Hybond-P membranes. The DM116 mAb was used to detect AP65 in transfected *T. foetus *(lane 2) and *T. vaginalis *(lane 4). No protein was detected in wt *T. foetus *(lane 1) and *T. foetus *transfected with the control plasmid (lane 3).

We next wanted to confirm that the episomal *ap65 *transcript seen in Figure 2 was translated. A 65-kDa band was readily detected by mAb 12G4 to AP65 in immunoblots of extracts of total proteins from *T. foetus *transfected with pBS *neo-ap65 *(Figure 3A, lane 2), and the protein had the same size as AP65 detected in total protein blots of *T. vaginalis *(lane 4). There were no detectable bands in either wild type *T. foetus *(lane 1) or *T. foetus *transfected with the control plasmid (lane 3). This result also confirms that the 12G4 mAb did not cross-react with any protein in blots of *T. foetus*.

Importantly, we further tested for the ability of episomal AP65 in *T. foetus *to bind to host cells using a ligand assay [15,25]. Figure 3B (lane 4) shows a typical result of a ligand assay using *T. vaginalis*, and mAb 12G4 used as a probe detected AP65 on blots of proteins that bound MS-74 VECs that were solubilized for electrophoresis and blotting. Likewise, AP65 from transfected parasites bound to MS-74 VECs (lane 2) and gave a band with the same M_r_. There were no detectable protein bands for wild-type *T. foetus *(lane 1) and *T. foetus *transfected with control plasmid (lane 3) handled identically in the ligand assay. These results show the ability of *T. foetus *transfectants to stably express functional AP65. Further, the lack of any immuno-crossreactivity with mAb is consistent with the absence of transcript (Figure 1B) and illustrates the absence of an AP65-like protein reactive with mAbs in *T. foetus*.

### AP65 expressed in *T. foetus* is compartmentalized to the surface and hydrogenosomes

**Figure 4:**
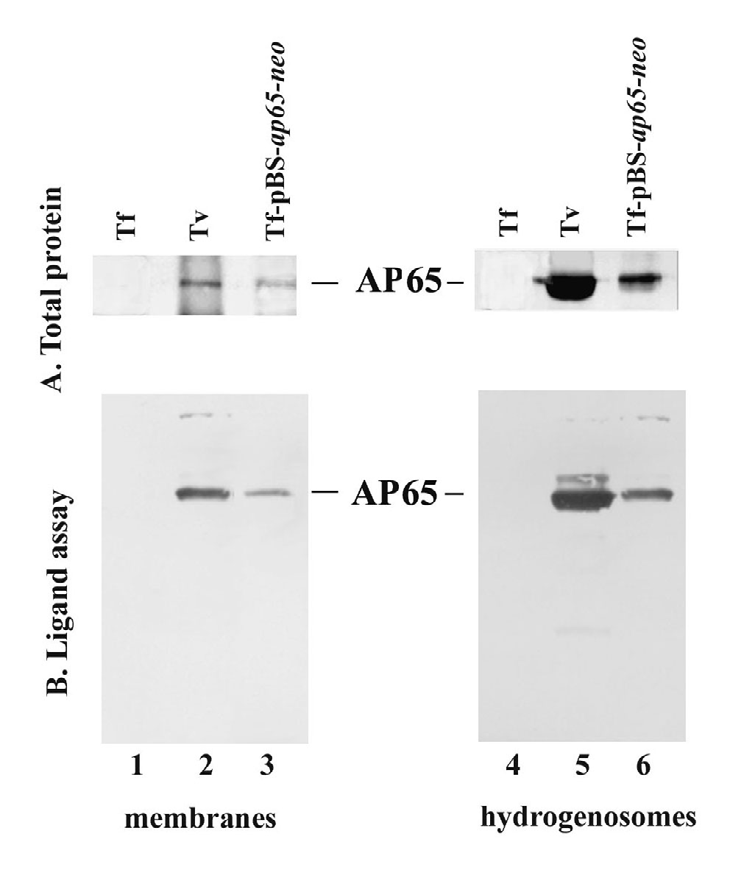
**Immunoblots with specific mAb 12G4 detecting AP65 immunoprecipitated from extracts of total trichomonad protein preparations (A) and of proteins after the ligand assay (B) derived from purified hydrogenosomes (lanes 1 through 3) and membrane fractions (lanes 4 through 6)**. AP65 was immunoprecipitated with mAb from protein extracts from hydrogenosomes and membrane fractions of *T. foetus *(*Tf*; lanes 1 and 4), *T. vaginalis *(*Tv*; lanes 2 and 5), and transfected *T. foetus *(Tf-pBS-*ap65-neo*; lanes 3 and 6). Immunoprecipitated AP65 was prepared as described in the Experimental design section and used in a ligand assay to monitor the amount of AP65 adhesin bound to MS-74 VECs as shown above in Figure 3.

Next, we performed cell membrane fractionation experiments to determine whether episomally-expressed AP65 in *T. foetus *transfectants localized to the surface membranes. Fractions enriched for membranes and hydrogenosome organelles were obtained by differential centrifugation, and the fractions were used for monitoring the presence of AP65 using a ligand assay. Figure 4A illustrates AP65 detected by mAb 12G4 in blots of total proteins from membranes and hydrogenosomes of *T. vaginalis *(lanes 2 and 5) and *T. foetus *transfected with the pBS-*ap65-neo *plasmid (lanes 3 and 6). As above, no bands were visualized on blots with total proteins of wild type *T. foetus *(lanes 1 and 4). We then used these fractions to perform a ligand assay, and as seen in Figure. 4B, both membrane and hydrogenosome fractions of *T. vaginalis *(lanes 2 and 5) and transfected *T. foetus *(lanes 3 and 6) had AP65 that bound to MS-74 VECs. Wild type *T. foetus *had no protein detected by mAb from the ligand assay (lanes 1 and 4).

### Immunofluorescence detects AP65 in transfectants on the surface and in hydrogenosomes

**Figure 5:**
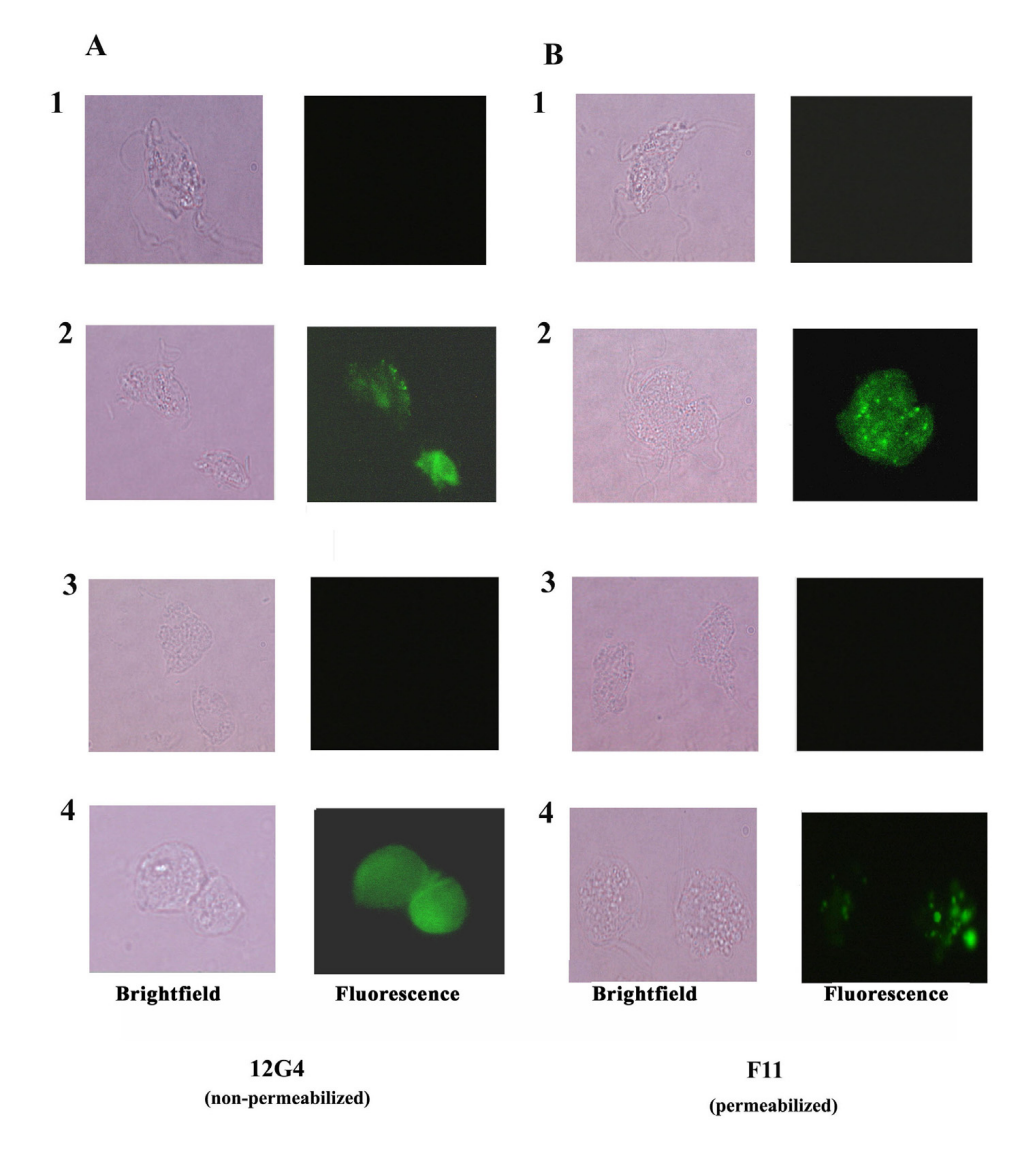
**Immunofluorescence and corresponding brightfield microscopy showing pBS-*ap65-neo *transfected *T. foetus *(Tf-pBS-*ap65-neo*) expressing AP65 on the surface of non-permeabilized trichomonads (A2) and in hydrogenosomes in permeabilized parasites (B2)**. The distinct patterns were obtained with the mAbs 12G4 for surface fluorescence and F11 for hydrogenosome fluorescence shown recently to detect the protein in respective compartments (Garcia *et al*., 2003). *T. vaginalis *fluorescence patterns (Tv; A4 and B4) were positive controls, and results are identical to those obtained recently (Garcia *et al*., 2003). No evidence of fluorescence was seen for wild type *T. foetus *(Tf) and for *T. foetus *transfected with control plasmid (Tf-pBS-*neo*). Control hybridoma supernatant lacking anti-AP65 mAb was unreactive with trichomonads.

It was then important to examine the possible placement and accessibility of episomal AP65 on the surface of *T. foetus*. Figure 5 presents immunofluorescence data with *T. vaginalis *showing mAb 12G4 detecting surface AP65 (A4) and mAb F11 reacting with hydrogenosomal AP65 (decarboxylating malic enzyme) (B4), data consistent with that shown in a recent report by us [16]. Importantly, as with the fractionation experiments performed above, mAb 12G4 detected AP65 on the surface of non-permeabilized transfected *T. foetus *(Figure 5A2), and mAb F11 was reactive with AP65 within hydrogenosomes of permeabilized trichomonads (Figure 5B2). The absence of any fluorescence for wild type *T. foetus *(A1 and B1) and *T. foetus *with control plasmid (A3 and B3) reinforced the specific binding of mAbs with episomal AP65 and reaffirmed results from Figures 3 and 4 above. The brightfield pictures are included to show the integrity of the trichomonads in the fluorescence experiments. These data further confirm that episomal AP65 is sorted both to the parasite surface and to hydrogenosome organelles, suggesting that the trafficking signals are present within the AP65 open reading frame and that *T. foetus *also possesses the protein compartmentalization machinery similar to that of *T. vaginalis*.

### Episomal expression of fusion AP65-HA and sorting to the parasite surface

**Figure 6:**
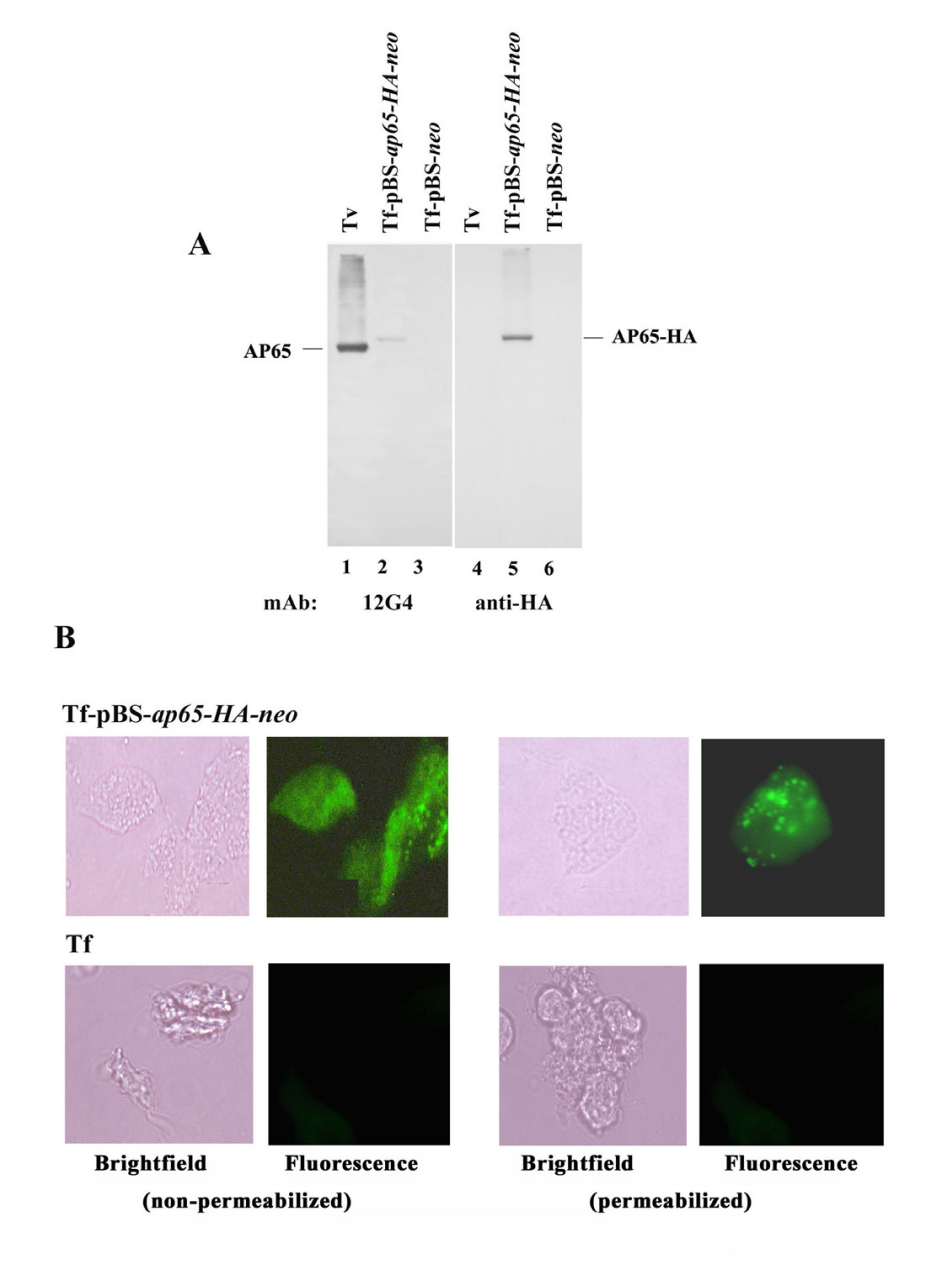
**Immunoblot detection of AP65-HA in *T. foetus *transfected with pBS-*ap65-HA-neo*, and immunofluorescence microscopy detecting AP65-HA protein on the surface (non-permeabilized) and in hydrogenosomes (permeabilized)**. (A) Duplicate immunoblots prepared from total proteins as above were probed with 12G4 mAb to AP65 (lanes 1 through 3) and anti-HA mAb (lanes 4 through 6). AP65 was readily detected by 12G4 in blots of *T. vaginalis *(lane 1) and *T. foetus *transfected with pBS-*ap65-HA-neo *(lane 2) but not *T. foetus *with control plasmid (lane 3). The anti-HA mAb detected the AP65-HA protein only in *T. foetus *transfected with pBS-*ap65-HA-neo *(lane 5). No protein was detectable by anti-HA mAb in blots of control *T. vaginalis *(lane 4) and *T. foetus *with the pBS-*neo *plasmid (lane 6). As an additional negative control for all immunoblots, no protein was detected in the absence of primary antibody or using control hybridoma supernatant. (B) Fluorescence is visualized using anti-HA mAb only in the *T. foetus *organisms transfected with the pBS-*ap65-HA-neo *plasmid but not the pBS-*neo *plasmid. Not unexpectedly, no fluorescence was seen using anti-HA mAb with *T. foetus *and with the use of control hybridoma supernatant lacking mAb to AP65. Finally, no fluorescence with anti-HA mAb was obtained using *T. vaginalis *organisms under identical experimental conditions.

Having established surface AP65 on transfected *T. foetus*, we next wanted to confirm surface placement using a different protein construct that would be detected by a distinct mAb. We, therefore, expressed the AP65-HA fusion protein, and immunoblot of total proteins of transfected *T. foetus *shows a band detected by the anti-HA mAb (Figure 6A, lane 5). As expected, the mAb 12G4 to AP65 detected a protein band in blots of total proteins of *T. vaginalis *(lane 1) and the pBS-*ap65-HA-neo *transfected *T. foetus *(lane 2). The M_r _of the fusion protein was higher than AP65, as expected. Also, no bands were apparent with any mAbs in *T. foetus *with control pBS-*neo *plasmid (lanes 3 and 6).

Finally, as shown in Figure 6B, immunofluorescence microscopy gave a positive reaction with anti-HA mAb with AP65-HA protein on the surface using non-permeabilized and in hydrogenosomes with permeabilized transfected (Tf-pBS-*ap65-neo*) trichomonads. The *T. foetus *transfected with the control plasmid (Tf-pBS-*neo*) was unreactive with anti-HA mAb and gave results identical to those shown for the wild type *T. foetus*. Finally, in data not shown, no fluorescence with anti-HA mAb was obtained using *T. vaginalis *organisms as controls under identical experimental conditions. These data using a different mAb to the fusion protein construct independently demonstrated the surface placement of episomal AP65 and AP65-HA on *T. foetus*.

### Episomally-expressed AP65 in *T. foetus* is related to enhanced adherence to VECs

**Figure 7:**
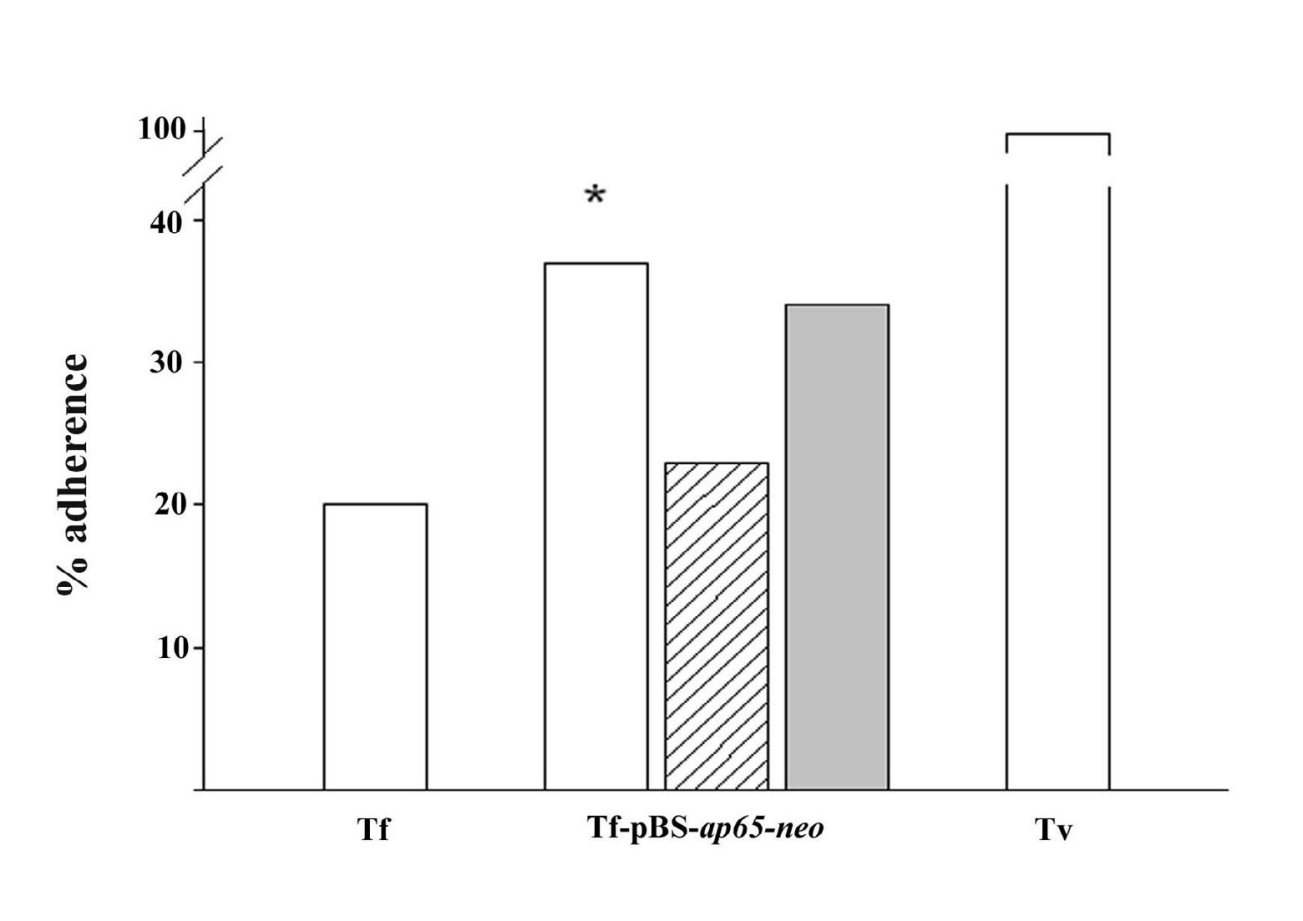
***T. foetus *transfected with pBS-*ap65-neo *(Tf-pBS-*ap65-neo*) displays enhanced levels of adherence to immortalized human MS-74 VECs compared to *T. foetus *(Tf) parasites**. The percent level of adherence was adjusted with that seen for *T. vaginalis *(Tv). *T. foetus *with control plasmid gave levels of adherence similar to *T. foetus *(data not shown). The enhanced adherence obtained with transfected *T. foetus *(Tf-pBS-*ap65-neo*) was inhibited by anti-AP65 IgG (hatched middle bar). The solid gray bar (right) represents inhibition by normal rabbit serum (NRS) control. The results are the average from four different experiments, and each experiment was carried out using quadruplicate samples. The statistical significance of the results is indicated by the asterisk above the bar graph.

Finally, we wanted to perform a functional adherence assay to determine if episomal, surface-expressed and host cell-binding AP65 on *T. foetus*, as shown collectively above, would enhance adherence of parasites to immortalized human MS-74 VECs. Figure 7 presents representative data showing that transfected parasites with pBS-*ap65-neo *were 40% more adherent to VECs when compared to the background level of adherence of wild type *T. foetus *(Tf). *T. foetus *with control plasmid gave lower adherence levels equal to wild type parasites (not shown). Importantly, the enhanced adherence was inhibited by 36 % in the presence of rabbit polyclonal anti-AP65 IgG antibody (striped bar), indicating specific AP65-mediated adherence, as before for *T. vaginalis *[15,16]. Control normal rabbit serum IgG did not inhibit adherence of the pBS-*ap65-neo *transfected *T. foetus *(shaded bar). Levels of adherence were compared with those of *T. vaginalis *(Tv) and normalized to 100%.

## Discussion

We have shown that trichomonads possess surface adhesins with functional diversity [26]. The adhesins are also enzymes in hydrogenosome organelles involved in energy generation. Although complex and multifactorial, the process of *T. vaginalis *adherence to human VECs is mediated in part by AP65, the surface protein that plays a major role in adherence [15,16]. In this study, we established the utility of the *T. foetus *bovine trichomonad as a heterologous expression model system. We feel that among the noteworthy findings of this report are the following: 1) We show the stable episomal expression of the *T. vaginalis *prominent *ap65 *gene (Figure 2). 2) The transcript is translated to form functional AP65 adhesin (Figure 3). 3) Episomal AP65 and fusion AP65-HA are compartmentalized to the surface and hydrogenosomes and are accessible to recognition by specific anti-AP65 and anti-HA mAbs (Figures 4 through 6). 4) AP65 on transfected *T. foetus *increased adherence to VECs compared to parasites transfected with control plasmid (Figure 7), and the enhanced binding was reduced with anti-AP65 antibodies, showing specific AP65 mediated VEC attachment.

The stable expression of *T. vaginalis *AP65 in transfected *T. foetus *allowed us to characterize via cell fractionation experiments the cellular location of the episomal protein. Remarkably, AP65 fractionated to the plasma membrane and hydrogenosomes (Figure 4). This fractionation data was reaffirmed by fluorescence experiments (Figure 5), and the ability to detect both surface AP65 and surface AP65-HA using distinct mAbs provides strong evidence for the trafficking of *T. vaginalis *proteins to distinct cellular compartments in the bovine trichomonad. These results indicate that machinery for recognition of the surface and organelle targeting sequences is similar among these phylogenetically-related trichomonad species. Importantly, the expression of AP65-HA now will permit future subclone analysis for identification of the protein region within AP65 directing surface channeling.

That surface AP65 on transfected *T. foetus *elevated levels of adherence to human VECs (Figure 7) is significant because human VECs are not the natural host cell for *T. foetus *[27,28]. The net increase in binding levels above *T. foetus *wild type controls were specific, as evidenced by the fact that anti-AP65 IgG reduced adherence to original control values. It is not inconceivable that the experimental conditions used here would not increase adherence by transfected *T. foetus *to those seen by *T. vaginalis*. One possible explanation is the fact that efficient and optimal adherence to VECs requires at least three additional adhesins, something that has been experimentally verified [15,16]. Alternatively, the copy number of episomal AP65 on *T. foetus *may not be optimal for production of amounts of adhesin needed for elevated adherence levels. Such a difference in copy number between AP65 on *T. vaginalis *and *T. foetus *(Fig. 3) may not be obvious based solely on fluorescence intensity, as this is not quantitative. In this regard, it may be that the equivalent protein of *T. foetus *decarboxylating malic enzyme is also surface expressed thereby interfering with sequestration of sufficient molecules of episomal AP65 for adherence. The future availability of specific antibodies to the *T. foetus *decarboxylating malic enzyme will be useful for testing this possibility. It is noteworthy that the *ap65 *gene and mAbs to AP65 did not cross-hybridize and immunoreact, respectively, with the equivalent decarboxylating malic enzyme gene and protein known to reside in hydrogenosomes of *T. foetus *[29,30]. These data also now indicate that there is significant nucleotide and amino acid sequence divergence between *T. vaginalis *and *T. foetus *in these equivalent genes and proteins. Importantly, except for the N-terminal sequence submitted to the GenBank (Accession number AAK55143.1), to our knowledge the entire decarboxylating malic enzyme gene of *T. foetus *has not been characterized [31]. Finally, not all episomal, surface-expressed AP65 molecules may be in the proper orientation to have adhesive function. Nonetheless, the significant increase in adherence attributable to episomal AP65 by transfected *T. foetus *once again underscores the role of AP65 in adherence by *T. vaginalis*.

This paper now provides evidence for the functional expression of a heterologous virulence factor in a related trichomonad species. This ability to express *T. vaginalis *or other foreign proteins using *T. foetus *as a model is particularly noteworthy, especially if an identical and/or equivalent virulence factor and property are not detectable within the bovine trichomonad. We feel that this approach is innovative and represents a significant advance for understanding the contribution of specific *T. vaginalis *virulence factors to overall mechanisms of trichomonal pathogenesis.

## Methods

### Parasite and cell cultures

*Trichomonas vaginalis *isolate T016 and *Tritrichomonas foetus *(02–97) were grown in Trypticase-yeast extract-maltose (TYM) medium supplemented with 10% heat-inactivated horse serum [32]. Immortalized human MS-74 VECs were grown as detailed recently by us [15,16] in D-MEM supplemented with 10% fetal bovine serum. VECs were grown at 37°C in a 5% CO_2 _atmosphere.

### Plasmid construction

The plasmid pBS-*ap65-neo *was constructed by cloning the coding region of AP65-3 in pBS-*FdHAHA-neo *(forward primer 5' GTCCAGCATATGATGCTCGCATCTTCA GTC-3' and reverse primer 5'-GTCCACGGTACCTTAGTAGAGTTGCTCGTATTC-3'). The parent plasmid pBS-*FdHAHA-neo *[33] was used by us recently [16]. The parent plasmid was partially digested and the 1.7-kb *ap65-3 *gene was cloned into the *NdeI *and *Asp718 *sites giving rise to pBS-*ap65-neo*. In order to generate AP65 with the HA tag, the stop codon of AP65 was mutated and cloned into pBS-*FdHAHA-neo *resulting in pBS-*ap65-HA-neo*. The new plasmids were confirmed by sequencing. Plasmid DNA for transfection was purified using maxi prep columns (Qiagen, Inc., Valencia, CA).

### Stable transfection and selection for G418 resistance

Transfection of *T. foetus *cells was carried out by electroporation [34]. Parasites at early logarithmic phase of growth were used for transfection. Briefly, 4 × 10^7 ^parasites were centrifuged at 1,800 rpm at 4°C, and the pellet was resuspended in 400 μl fresh TYM before transferring into a 4-mm gap cuvette (BTX^®^, Genetronics, Inc., San Diego, CA) with 25 μg of plasmid DNA. Electroporation was performed at 320 V, 1000 microfarads and 725 ohms using the ECM 630 Electro cell manipulator (BTX^®^). Following the pulse, cells were placed on ice for 10 min and transferred into two T25 flasks with 50 ml of fresh TYM-serum medium. The cells were grown free of drug for 24 h followed by the addition of Geneticin (Invitrogen-Life Technologies, Carlsbad, CA) at 100 μg ml^-1^. Single cells were cloned using soft-agar plates [35]. The DNA was isolated from single cell cultures using DNAZol (Invitrogen) and further purified by phenol-chloroform extraction. The presence of plasmid in single cell clones was confirmed by PCR amplification of the *neo *gene. The sense 5' GATCGGTACCATGATTGATTGAACA AGATGGATTG-3', and antisense 5'-CTTTAGACCAAGTTCGTGTCAGAAGAACT CGTCAAG-3' primers were used in the PCR reaction.

### RNA isolation and RT-PCR analysis

Total RNA was isolated using the Trizol reagent (Invitrogen). Briefly, 1 μg of total RNA was reverse transcribed using SuperScript II RNase H^- ^Reverse Transcriptase (Invitrogen) in a reaction volume of 20 μl. One μl of the reverse transcribed cDNA was used as template for the PCR amplification. The *ap65 *primer sequences used for amplifications were *ap65*-sense 5'-CAGTCAGTCGACCAGTTAGATATGGGTACAGAC-3' and *ap65*-antisense 5'-GTGACAGGATCCCGCTCGCAGTTAGCGCATGTAG-3'. The *α-tubulin *gene primers were sense 5'-ACTCTGCTGCCTCGAGCACGGTATC-3' and antisense 5'-GAAATGACTGGTGCATAAGAGC-3'. The common annealing temperature for the primers of both genes allowed for the *ap65 *and *α-tubulin *primers to be included in the same PCR reaction.

### Immunoblot detection of episomally-expressed AP65

Total protein from parasites was obtained as before using trichloroacetic acid (TCA) precipitation [36] and separated on sodium dodecylsulfate-polyacrylamide gel electrophoresis (SDS-PAGE) [37] prior to blotting onto Hybond-P membranes (Amersham Pharmacia Biotech, Pisscataway, NJ) for immunoblot detection with mAb 12G4 to AP65 and anti-HA mAb. The mAbs and epitope reactivity were described recently [15]. Following blot reactivity with the mAbs, the bands were visualized by the chemiluminescence assay using horseradish peroxidase as the color developer (BioRad Laboratories, Hercules, CA).

### Sub-cellular fractionation

The hydrogenosomes were isolated as recently detailed [15]. Briefly, 1 × 10^8 ^trichomonads were homogenized in buffer (250 mM sucrose, 20 mM KCL, 10 mM KH_2_PO_4_, 5 mM MgCl_2 _and 20 mM Tris-HCl, pH 7.0). The homogenate was centrifuged to sediment nuclei and costae at 1,500 g for 10 min at 4°C. The supernatant was then transferred to a new tube and centrifuged at 12,000 × g for 10 min. The sediment was then washed once more with homogenization buffer and was highly enriched for hydrogenosomes, which was confirmed biochemically as recently described [16]. The sediment was resuspended in the buffer and stored at -70°C. Membrane isolation was also carried out as described recently [38]. *T. foetus *(1 × 10^8^) were resuspended in phosphate-buffered saline (PBS), sonicated, and subjected to low-speed centrifugation at 500 × g for 15 min at 4°C. The resulting supernatant was further fractionated by sequential differential centrifugations of 1,500 × g, 10,000 × g, and 100,000 × g for 30 min at 4°C. The supernatant was used for protein analysis.

### Ligand assay to determine the amounts of adhesins on the surface

The ligand assay to detect adhesins that bind to host cell surfaces was carried out as before [25]. Briefly, after fixation of the MS-74 VECs with glutaraldehyde, 10^6 ^epithelial cells were incubated with a trichomonal detergent extract derived from 2 × 10^7 ^solubilized parasites. After incubation, cells were vigorously washed to remove unbound and loosely-associated trichomonad proteins. Cells were boiled in electrophoresis dissolving buffer to elute the proteins bound to the MS-74 VEC followed by SDS-PAGE. The gels were further stained with Coomassie Brilliant blue for visualization, and duplicate gels were blotted onto Hybond-P membranes for immunoblot analysis using the mAbs as probes.

### Immunoprecipitation studies

AP65 from the membranes and hydrogenosomes derived from the fractionation experiments was immunoprecipitated using mAb DM116 [15]. A standard immunoprecipitation protocol was used [39]. Briefly, the fractions (0.5 mg) prepared as mentioned above, were mixed with 5.0 μg of mAb DM116 for 4 h at 4°C. The extract and mAb were mixed on a rotating platform for 3 h with Protein A-Sepharose beads. The beads were washed three times with PBS, and samples were eluted by boiling for 10 min in SDS dissolving buffer. Eluted proteins were resolved by SDS-PAGE, and immunoblot analyses and ligand assays were performed as described above.

### Immunofluorescence detection of AP65 on the surface

Immunofluorescence of AP65 on the surface and in hydrogenosomes of trichomonads was carried out using a modification of a recently-described procedure [15]. Briefly, 1 × 10^6 ^logarithmic-phase organisms were washed twice with cold PBS and fixed with 4 % paraformaldehyde for 10 min at RT. Fixed cells were washed in PBS and permeabilized with 1% NP-40 for 45 min at RT. Trichomonads were then blocked with 5% BSA for 1 h at RT prior to incubation for an additional 1 h at RT with hybridoma supernatants of mAb 12G4 (1:100) and anti-HA mAb F11 (1:1000). Parasites were washed with PBS and incubated for 1 h at 37°C with fluorescein isothiocyanate-conjugated anti-mouse IgG (Sigma) diluted 1:100. Finally, parasites were washed twice with PBS and observed under 1000× magnification using the Olympus BX41 microscope.

### Adherence assay

Immortalized human MS-74 VECs [40] were used for the adherence assay as recently described [15]. Briefly, 2 × 10^4 ^MS-74 VECs were seeded onto individual wells of 96-well Costar flat bottom plates (Corning Inc., Corning, NY) and grown for 24 h in DMEM supplemented with 10 % fetal bovine serum (FBS). VECs were then washed twice with a medium mixture of DMEM:TYM (2:1; v/v) without serum. Trichomonads were labeled with [^3^H] thymidine for 18 h, washed three times with DMEM-TYM and resuspended. Then, 4 × 10^5 ^tritium-labeled parasites were added to the individual wells of the 96-well plate with a confluent MS-74 VEC monolayer and incubated for 30 min at 37°C. Cells were then washed thoroughly with the DMEM-TYM. Individual wells were placed in mini-scintillation vials and radioactivity was measured. The assay was performed with quadruplicate samples.

### Reproducibility of experiments

Unless otherwise stated in the text, all experiments were performed numerous times and no less than on four different occasions.

## Abbreviations

AP65, adhesin protein Mr 65-kDa; DMEM, Dulbecco's minimum essential medium; HA, hemagglutinin; kb, kilobase; M_r_, relative molecular weight; pBS-*ap65-neo*, plasmid construct with the *ap65 *and *neomycin *genes; Tf, *Tritrichomonas foetus*; Tv, *Trichomonas vaginalis*; TYM, trypticase-yeast extract-maltose; VECs, vaginal epithelial cells.

## Authors' contributions

ASK carried out the design of the study, performed transfections, Western blots, immunofluorescence, adherence assays and drafted the manuscript. VM constructed the plasmids, performed the Northern analysis, RT-PCR and wrote parts of the manuscript. JFA participated in the design of the experiments, offered suggestions during the experiments, and helped to draft the manuscript. All authors read and approved the final manuscript.

## References

[B1] Kassai T, Cordero de Campillo M, Euzeby J, Gafar S, Hiepe T, Himonas CA (1988). Standardized nomenclature of animal parasitic diseases (SNOAPAD). Vet Parasitol.

[B2] Weinstock H, Berman S, Cates W (2004). Sexually transmitted disease among American youth: incidence and prevalence estimates, 2000. Perspect Sex Reprod Health.

[B3] Sorvillo F, Kovacs A, Kerndt P, Stek A, Muderspach L, Sanchez-Keeland L (1998). Risk factors for trichomoniasis among women with human immunodeficiency virus (HIV) infection at a public clinic in Los Angeles County, California: implications for HIV prevention. Am J Trop Med Hyg.

[B4] El-Shazly AM, El-Naggar HM, Soliman M, El-Negeri M, El-Nemr HE, Handousa AE, Morsy TA (2001). A study on *Trichomonas vaginalis *and female infertility. J Egypt Soc Parasitol.

[B5] Sherman KJ, Chow WH, Daling JR, Weiss NS (1988). Sexually transmitted diseases and the risk of tubal pregnancy. J Reprod Med.

[B6] Moodley P, Wilkinson D, Connolly C, Moodley J, Sturm AW (2002). *Trichomonas vaginalis *is associated with pelvic inflammatory disease in women infected with human immunodeficiency virus. Clin Infect Dis.

[B7] Hobbs MM, Kazembe P, Reed AW, Miller WC, Nkata E, Zimba D, Daly CC, Chakraborty H, Cohen MS, Hoffman I (1999). *Trichomonas vaginalis *as a cause of urethritis in Malawian men. Sex Transm Dis.

[B8] Sorvillo F, Smith L, Kerndt P, Ash L (2001). *Trichomonas vaginalis*, HIV, and African-Americans. Emerg Infect Dis.

[B9] Cotch MF, Pastorek JG, Nugent RP, Hillier SL, Gibbs RS, Martin DH, Eschenbach DA, Edelman R, Carey JC, Regan JA, Krohn MA, Klebanoff MA, Rao AV, Rhoads GG (1997). *Trichomonas vaginalis *associated with low birth weight and preterm delivery. The Vaginal Infections and Prematurity Study Group. Sex Transm Dis.

[B10] Viikki M, Pukkala E, Nieminen P, Hakama M (2000). Gynaecological infections as risk determinants of subsequent cervical neoplasia. Acta Oncol.

[B11] Bakare RA, Ashiru JO, Adeyemi-Doro FA, Ekweozor CC, Oni AA, Okesola AO, Adebayo JA (1999). Non-gonococcal urethritis (NGU) due to *Trichomonas vaginalis *in Ibadan. West Afr J Med.

[B12] Bennett JR, Barnes WG, Coffman S (1989). The emergency department diagnosis of Trichomonas vaginitis. Ann Emerg Med.

[B13] Alderete JF, Garza GE (1988). Identification and properties of *Trichomonas vaginalis *proteins involved in cytadherence. Infect Immun.

[B14] Alderete JF, Garza GE (1985). Specific nature of *Trichomonas vaginalis *parasitism of host cell surfaces. Infect Immun.

[B15] Garcia AF, Chang TH, Benchimol M, Klumpp DJ, Lehker MW, Alderete JF (2003). Iron and contact with host cells induce expression of adhesins on surface of *Trichomonas vaginalis*. Mol Microbiol.

[B16] Mundodi V, Kucknoor AS, Klumpp DJ, Chang TH, Alderete JF (2004). Silencing the ap65 gene reduces adherence to vaginal epithelial cells by *Trichomonas vaginalis*. Mol Microbiol.

[B17] Anderson ML, BonDurant RH, Corbeil RR, Corbeil LB (1996). Immune and inflammatory responses to reproductive tract infection with *Tritrichomonas foetus *in immunized and control heifers. J Parasitol.

[B18] Felleisen RS (1999). Host-parasite interaction in bovine infection with *Tritrichomonas foetus*. Microbes Infect.

[B19] da Silva NS, Dias Filho BP, de Souza W (1999). Identification and localization of an adhesin on the surface of *Tritrichomonas foetus*. Parasitol Res.

[B20] Shaia CI, Voyich J, Gillis SJ, Singh BN, Burgess DE (1998). Purification and expression of the Tf190 adhesin in *Tritrichomonas foetus*. Infect Immun.

[B21] Singh BN, Lucas JJ, Beach DH, Shin ST, Gilbert RO (1999). Adhesion of *Tritrichomonas foetus *to bovine vaginal epithelial cells. Infect Immun.

[B22] Singh BN, Lucas JJ, Hayes GR, Kumar I, Beach DH, Frajblat M, Gilbert RO, Sommer U, Costello CE (2004). *Tritrichomonas foetus *induces apoptotic cell death in bovine vaginal epithelial cells. Infect Immun.

[B23] O'Brien JL, Lauriano CM, Alderete JF (1996). Molecular characterization of a third malic enzyme-like AP65 adhesin gene of *Trichomonas vaginalis*. Microb Pathog.

[B24] Gilbert RO, Elia G, Beach DH, Klaessig S, Singh BN (2000). Cytopathogenic effect of *Trichomonas vaginalis *on human vaginal epithelial cells cultured in vitro. Infect Immun.

[B25] Arroyo R, Engbring J, Alderete JF (1992). Molecular basis of host epithelial cell recognition by *Trichomonas vaginalis*. Mol Microbiol.

[B26] Alderete JF, Millsap KW, Lehker MW, Benchimol M (2001). Enzymes on microbial pathogens and *Trichomonas vaginalis *: molecular mimicry and functional diversity. Cell Microbiol.

[B27] Burgess DE, McDonald CM (1992). Analysis of adhesion and cytotoxicity of *Tritrichomonas foetus *to mammalian cells by use of monoclonal antibodies. Infect Immun.

[B28] Hodgson JL, Jones DW, Widders PR, Corbeil LB (1990). Characterization of *Tritrichomonas foetus *antigens by use of monoclonal antibodies. Infect Immun.

[B29] Kulda J (1999). Trichomonads, hydrogenosomes and drug resistance. Int J Parasitol.

[B30] Steinbuchel A, Muller M (1986). Anaerobic pyruvate metabolism of *Tritrichomonas foetus *and *Trichomonas vaginalis *hydrogenosomes. Mol Biochem Parasitol.

[B31] Land KM, Clemens DL, Johnson PJ (2001). Loss of multiple hydrogenosomal proteins associated with organelle metabolism and high-level drug resistance in trichomonads. Exp Parasitol.

[B32] Diamond LS (1957). The establishment of various trichomonads of animals and man in axenic cultures. J Parasitol.

[B33] Land KM, Delgadillo MG, Johnson PJ (2002). In vivo expression of ferredoxin in a drug resistant trichomonad increases metronidazole susceptibility. Mol Biochem Parasitol.

[B34] Tsai CD, Liu HW, Tai JH (2002). Characterization of an iron-responsive promoter in the protozoan pathogen *Trichomonas vaginalis*. J Biol Chem.

[B35] Delgadillo MG, Liston DR, Niazi K, Johnson PJ (1997). Transient and selectable transformation of the parasitic protist *Trichomonas vaginalis*. Proc Natl Acad Sci U S A.

[B36] Alderete JF (1983). Identification of immunogenic and antibody-binding membrane proteins of pathogenic *Trichomonas vaginalis*. Infect Immun.

[B37] Dias Filho, Benchimol M, Andrade AF, Angluster J, De Souza W (1999). Purification and immunocytochemical localization of neuraminidase from *Tritrichomonas foetus*. Parasitology.

[B38] Laemmli UK (1970). Cleavage of structural proteins during the assembly of the head of bacteriophage T4. Nature.

[B39] Kessler SW (1975). Rapid isolation of antigens from cells with a staphylococcal protein A-antibody adsorbent: parameters of the interaction of antibody-antigen complexes with protein A. J Immunol.

[B40] Klumpp DJ, Forrestal SG, Karr JE, Mudge CS, Anderson BE, Schaeffer AJ (2002). Epithelial differentiation promotes the adherence of type 1-piliated *Escherichia coli *to human vaginal cells. J Infect Dis.

